# A multi-institutional meningioma MRI dataset for automated multi-sequence image segmentation

**DOI:** 10.1038/s41597-024-03350-9

**Published:** 2024-05-15

**Authors:** Dominic LaBella, Omaditya Khanna, Shan McBurney-Lin, Ryan Mclean, Pierre Nedelec, Arif S. Rashid, Nourel hoda Tahon, Talissa Altes, Ujjwal Baid, Radhika Bhalerao, Yaseen Dhemesh, Scott Floyd, Devon Godfrey, Fathi Hilal, Anastasia Janas, Anahita Kazerooni, Collin Kent, John Kirkpatrick, Florian Kofler, Kevin Leu, Nazanin Maleki, Bjoern Menze, Maxence Pajot, Zachary J. Reitman, Jeffrey D. Rudie, Rachit Saluja, Yury Velichko, Chunhao Wang, Pranav I. Warman, Nico Sollmann, David Diffley, Khanak K. Nandolia, Daniel I Warren, Ali Hussain, John Pascal Fehringer, Yulia Bronstein, Lisa Deptula, Evan G. Stein, Mahsa Taherzadeh, Eduardo Portela de Oliveira, Aoife Haughey, Marinos Kontzialis, Luca Saba, Benjamin Turner, Melanie M. T. Brüßeler, Shehbaz Ansari, Athanasios Gkampenis, David Maximilian Weiss, Aya Mansour, Islam H. Shawali, Nikolay Yordanov, Joel M. Stein, Roula Hourani, Mohammed Yahya Moshebah, Ahmed Magdy Abouelatta, Tanvir Rizvi, Klara Willms, Dann C. Martin, Abdullah Okar, Gennaro D’Anna, Ahmed Taha, Yasaman Sharifi, Shahriar Faghani, Dominic Kite, Marco Pinho, Muhammad Ammar Haider, Michelle Alonso-Basanta, Javier Villanueva-Meyer, Andreas M. Rauschecker, Ayman Nada, Mariam Aboian, Adam Flanders, Spyridon Bakas, Evan Calabrese

**Affiliations:** 1 Department of Radiation Oncology, Duke University Medical Center, Durham, NC USA; 2 Department of Neurosurgery, Thomas Jefferson University, Philadelphia, PA USA; 3 Center for Intelligent Imaging (ci2), Department of Radiology & Biomedical Imaging, University of California San Francisco (UCSF), San Francisco, CA USA; 4 Yale University, New Haven, CT USA; 5 Department of Radiation Oncology, Perelman School of Medicine, University of Pennsylvania, Philadelphia, PA USA; 6 University of Missouri, Columbia, MO USA; 7 Division of Computational Pathology, Department of Pathology and Laboratory Medicine, School of Medicine, Indiana University, Indianapolis, IN USA; 8 Center for Data-Driven Discovery in Biomedicine (D3b), The Children’s Hospital of Philadelphia, Philadelphia, PA USA; 9 Department of Neurosurgery, The Children’s Hospital of Philadelphia, Philadelphia, PA USA; 10 Department of Neurosurgery, Perelman School of Medicine, University of Pennsylvania, Philadelphia, PA USA; 11 Helmholtz AI, Helmholtz Munich, Neuherberg, Germany; 12 Department of Computer Science, TUM School of Computation, Information and Technology, Technical University of Munich, Munich, Germany; 13 TranslaTUM - Central Institute for Translational Cancer Research, Tech nical University of Munich, Munich, Germany; 14 Department of Diagnostic and Interventional Neuroradiology, School of Medicine, Klinikum rechts der Isar, Technical University of Munich, Munich, Germany; 15 University of Zurich, Zurich, Switzerland; 16 Department of Radiology, University of California San Diego, San Diego, CA USA; 17 Department of Radiology, Cornell University, Ithaca, NY USA; 18 Department of Radiology, Northwestern University, Evanston, IL USA; 19 Duke University Medical Center, School of Medicine, Durham, NC USA; 20 Department of Diagnostic and Interventional Radiology, University Hospital Ulm, Ulm, Germany; 21 TUM-Neuroimaging Center, Klinikum rechts der Isar, Technical University of Munich, Munich, Germany; 22 Fort Worth, TX USA; 23 Department of Diagnostic and Interventional Radiology, All India Institute of Medical Sciences, Rishikesh, India; 24 Department of Neuroradiology, Washington University, St. Louis, MO USA; 25 University of Rochester Medical Center, Rochester, NY USA; 26 Faculty of Medicine, Jena University Hospital, Friedrich Schiller University Jena, Jena, Germany; 27 vRad (Radiology Partners), Minneapolis, MN USA; 28 Ross University School of Medicine, Bridgetown, Barbados; 29 Department of Radiology, New York University Grossman School of Medicine, New York, NY USA; 30 Department of Radiology, Arad Hospital, Tehran, Iran; 31 Department of Radiology, Faculty of Medicine, University of Ottawa, Ottawa, Canada; 32 Department of Neuroradiology, JDMI, University of Toronto, Toronto, TO Canada; 33 Department of Radiology, Azienda Ospedaliero Universitaria of Cagliari-Polo di Monserrato, Cagliari, Italy; 34 Department of Radiology, Leeds General Infirmary, Leeds, UK; 35 Ludwig Maximilians University, Munich, Bavaria Germany; 36 Rush University Medical Center, Chicago, IL USA; 37 Department of Neurosurgery, University Hospital of Ioannina, Ioannina, Greece; 38 Department of Neuroradiology, University Hospital Essen, Essen, North Rhine-Westphalia Germany; 39 Egyptian Ministry of Health, Cairo, Egypt; 40 Department of Radiology, Kasr Alainy, Cairo University, Cairo, Egypt; 41 Faculty of Medicine, Medical University of Sofia, Sofia, Bulgaria; 42 Department of Radiology, Perelman School of Medicine, University of Pennsylvania, Philadelphia, PA USA; 43 Department of Radiology, American University of Beirut Medical center, Beirut, Lebanon; 44 Radiology Department, King Faisal Medical City, Abha, Saudi Arabia; 45 Department of Diagnostic and Interventional Radiology, Cairo University, Cairo, Egypt; 46 Department of Radiology and Medical Imaging, University of Virginia Health, Charlottesville, VA USA; 47 Department of Radiology and Radiologic Sciences, Vanderbilt University Medical Center, Nashville, TN USA; 48 Faculty of Medicine, Hamburg University, Hamburg, Germany; 49 Neuroimaging Unit, ASST Ovest Milanese, Legnano, Milan, Italy; 50 University of Manitoba, Winnipeg, Manitoba Canada; 51 Department of Radiology, School of Medicine, Iran University of Medical Sciences, Tehran, Iran; 52 Radiology Informatics Lab, Department of Radiology, Mayo Clinic, Rochester, MN USA; 53 Department of Radiology, University Hospitals Bristol and Weston NHS Foundation Trust, Bristol, United Kingdom; 54 Department of Radiology, University of Texas Southwestern Medical Center, Dallas, TX USA; 55 CMH Lahore Medical College, Lahore, Pakistan; 56 Department of Radiology, Children’s Hospital of Philadelphia (CHOP), Philadelphia, PA USA; 57 Department of Radiology, Thomas Jefferson University, Philadelphia, PA USA; 58 Department of Neurological Surgery, School of Medicine, Indiana University, Indianapolis, IN USA; 59 Department of Radiology and Imaging Sciences, School of Medicine, Indiana University, Indianapolis, IN USA; 60 Department of Radiology, Duke University Medical Center, Durham, NC USA

**Keywords:** Translational research, CNS cancer, Brain, Databases, Brain imaging

## Abstract

Meningiomas are the most common primary intracranial tumors and can be associated with significant morbidity and mortality. Radiologists, neurosurgeons, neuro-oncologists, and radiation oncologists rely on brain MRI for diagnosis, treatment planning, and longitudinal treatment monitoring. However, automated, objective, and quantitative tools for non-invasive assessment of meningiomas on multi-sequence MR images are not available. Here we present the BraTS Pre-operative Meningioma Dataset, as the largest multi-institutional expert annotated multilabel meningioma multi-sequence MR image dataset to date. This dataset includes 1,141 multi-sequence MR images from six sites, each with four structural MRI sequences (T2-, T2/FLAIR-, pre-contrast T1-, and post-contrast T1-weighted) accompanied by expert manually refined segmentations of three distinct meningioma sub-compartments: enhancing tumor, non-enhancing tumor, and surrounding non-enhancing T2/FLAIR hyperintensity. Basic demographic data are provided including age at time of initial imaging, sex, and CNS WHO grade. The goal of releasing this dataset is to facilitate the development of automated computational methods for meningioma segmentation and expedite their incorporation into clinical practice, ultimately targeting improvement in the care of meningioma patients.

## Background & Summary

Meningiomas are the most common primary intracranial tumor in adults and can result in significant morbidity and mortality for affected patients^1,2^. Most meningiomas (∼80%) are fifth edition CNS World Health Organization (WHO) grade 1 benign tumors and are typically well controlled with observation, surgical resection, and/or radiation therapy^3,4^. However, higher grade meningiomas (CNS WHO grades 2 and 3) are associated with significantly higher morbidity and mortality rates and often recur despite optimal management^3,5^. Currently there is no reliable noninvasive method for identifying meningioma CNS WHO grade, assessing aggressiveness, or predicting recurrence and survival. Traditional MRI features used by clinicians to guide treatment strategy, such as meningioma size, or degree of surrounding edema, may not represent CNS WHO grade or expected clinical course^2^. As such, there is a need for improved radiographic assessment of meningiomas, which can help guide patient-specific treatment strategies.

Automated tumor segmentation on brain magnetic resonance imaging (MRI) has matured into a clinically viable tool that can provide objective assessments of tumor volume and can assist in surgical planning, radiotherapy planning, and treatment response assessment. However, to date, most brain tumor segmentation studies have focused exclusively on gliomas, despite the fact that meningiomas are more common, accounting for over a third of all intracranial tumors^6–8^. Meningiomas, while typically more circumscribed than gliomas, provide additional technical challenges for segmentation given their extra-axial location and propensity for skull-base involvement^2^. In addition, unlike other intracranial tumors, meningiomas are commonly diagnosed by imaging alone, which increases the importance of MRI for treatment planning.

The BraTS organization has conducted large scale international automated segmentation challenges focused on gliomas since 2012^7,8^. The initial 2012 BraTS glioma dataset consisted of 35 training and 15 testing cases. Each case consisted of co-registered multi-sequence pre- and postcontrast MR images with associated manually annotated tumor sub-compartment labels^7^. Recently, the 2021 BraTS glioma challenge described a multi-society effort across RSNA, ASNR, and MICCAI, that resulted in a dataset of >2,000 cases split across 1251 training cases, 219 validation cases, and 570 testing cases^9^. Due to the successful historic efforts focused on automated segmentation of glioma^7,8^, in 2023, the BraTS organization decided to compliment the adult glioma segmentation with a cluster of challenges. This cluster includes a dedicated meningioma segmentation challenge, the “Brain Tumor Segmentation Challenge 2023: Intracranial Meningioma”^10^.

Here we present the BraTS Pre-operative Meningioma Dataset, which is the largest known publicly available multi-institutional dataset of meningioma multi-sequence MR images to date^11,12^. The BraTS Pre-operative Meningioma Dataset includes 1,141 publicly available, pre-processed, multi-sequence MR images from six different academic medical centers with manually annotated sub-compartment tumor labels, basic demographic data, and CNS WHO grade when available. The purpose of this dataset is to facilitate the development of automated multi-compartment brain MRI segmentation algorithms for intracranial meningiomas. Segmentation algorithms developed using these data will allow objective assessment of tumor volume for surgical and radiotherapy planning and will serve as a starting point for future studies focused on identifying meningioma CNS WHO grade, assessing aggressiveness, and predicting risk of recurrence based on MRI findings alone. This manuscript describes the data collection, curation, and segmentation process for the BraTS Pre-operative Meningioma Dataset.

## Methods

### Study population

The study population consisted of adult patients diagnosed with intracranial meningioma of any CNS WHO grade or subtype either by imaging or histopathology following resection or biopsy^4^. Participants were retrospectively identified from six different academic medical centers: Duke University, Yale University, Thomas Jefferson University, University of California San Francisco, University of Missouri, and University of Pennsylvania. The specific case inclusion methods (pathologic, clinical/radiologic, or both) and case collection methods (i.e., random, consecutive) were chosen by each participating site independently, often on the basis of pre-existing curated datasets. Strict requirements on participant inclusion were not imposed to reduce barriers to data sharing. All participating sites had institutional review board (IRB) approval. A waiver for informed consent was provided by each institution’s respective IRB. All image data were anonymized, and faces were digitally removed to prevent facial reconstruction.

### Imaging data

Imaging data included pre-operative and pre-treatment multi-sequence MR images of the brain with corresponding expert annotated tumor sub-compartments. Multi-sequence MR images included pre-contrast T1-weighted, post-contrast T1-weighted, T2-weighted, and T2/FLAIR-weighted imaging. Exclusion criteria included lack of visible tumor on the skull-stripped MRI or the presence of any intracranial tumor that was not radiographically or pathologically consistent with meningioma (including cases of neurofibromatosis type 2 with intracranial schwannomas). Imaging parameters including field strength, echo/repetition time, slice resolution, and slice thickness varied considerably between and within sites and documentation of these variables were not required for data contribution, with the intention of reducing barriers to data sharing. The naming convention used for the anonymized case IDs was “BraTS-MEN-00XXX-00N”. In this format, “XXX” represents a unique identifier for each patient, and “00 N” indicates the interval case number of the respective pre-operative study for that particular patient. For example, if a patient underwent three pre-operative MRI studies that were included in the dataset, these would be labeled as “−001” for the first chronological case, “−002” for the second case, and “−003” for the third case, respectively, following the unique patient identifier “00XXX”. This system ensures that each case is distinctly identified, not just by the patient to whom it belongs but also by the order in which the studies were performed.

### Data splits

A total of 1,424 individual MRI exams from 1,344 different patients were included in the final BraTS Pre-operative Meningioma Dataset. These were divided into a training set (1,000/1,424, 70%), a validation set (141/1,424, 10%), and a private hold-out testing set (283/1,424, 20%). Splits were random but stratified by site and by patient such that interval multi-sequence MR images from each individual patient, as denoted by “00 N”, were assigned to a single data split. Training and validation data are being made publicly available, as part of this manuscript, while the testing data is private to allow unbiased ongoing evaluation of new segmentation methods through the synapse.org platform^13^.

### Clinical data

Clinical-pathologic information including patient age at the time of imaging, sex, and CNS WHO grade if available, were obtained from the respective electronic medical records at each institution. The age range was 14–96 years (including private testing set data) and 14–96 years in the publicly available data. The male to female ratio was 398:1,007 (including private testing set data) and 313:816 in the publicly available data. CNS WHO grade was available in 1,010 of the 1,424 total cases (including private testing set data) and in 800 of the 1,141 publicly available cases. Aggregate clinical-pathologic case-level data are provided in Table 1 (including private testing set data). Individual case-level data for the publicly available training and validation cases are freely provided on the Synapse data repository^14^.

**Table 1:** Basic clinical and demographic data for the BraTS Pre-operative Meningioma Dataset cohort including the private test set data for cases with available patient demographic data.

	Total	Training Set	Validation Set	Testing Set	Age (Median; Min-Max)	Male: Female	CNS WHO Grade 1	CNS WHO Grade 2	CNS WHO Grade 3
All Sites	1,424	1000 (70%)	141 (10%)	283 (20%)	61 (14–96)	398: 1,007	754	227	29
DUKE	452	315 (70%)	46 (10%)	91 (20%)	65 (19–96)	115: 337	115	24	2
JEFF	338	236 (70%)	34 (10%)	68 (20%)	60 (19–90)	114: 224	292	37	9
YALE	230	160 (70%)	23 (10%)	47 (20%)	57 (20–92)	69: 161	157	71	2
MISS	181	132 (73%)	16 (9%)	33 (18%)	64 (14–89)	38: 143	171	10	0
UCSF	180	126 (70%)	18 (10%)	35 (19%)	58 (15–88)	41: 119	19	41	16
PENN	44	31 (70%)	4 (9%)	9 (20%)	59 (21–87)	21: 23	0	44	0

Site abbreviations are as follows: DUKE (Duke University); JEFF (Thomas Jefferson University); YALE (Yale University); MISS (Missouri University); UCSF (University of California San Francisco); PENN (University of Pennsylvania).

### Image data pre-processing

All MRI data underwent standardized image pre-processing steps including conversion from Digital Imaging and Communications in Medicine (DICOM) format to Neuroimaging Informatics Technology Initiative (NIfTI) format, co-registration of individual image series to a canonical anatomical brain (i.e., the SRI24 atlas^15^ space), including uniform 1 mm^3^ isotropic resampling, and automated skull-stripping using a deep convolutional neural network approach^16^. These image pre-processing steps were implemented in the open-source and publicly available Federated Tumor Segmentation (FeTS) tool^17^, which is the same tool that facilitated the largest brain glioblastoma study to date^18^. It should be noted that meningioma can extend through the skull and/or skull-base foramina and that any extra-cranial portions of tumors were implicitly excluded by the skull-stripping process^2^. Despite this limitation, skull-stripping was included in the pre-processing to preserve patient anonymity (by preventing potential face reconstruction) and to ensure consistency with the other BraTS 2023 challenges.

### Defining meningioma sub-compartments on MRI

**Fig. 1:**
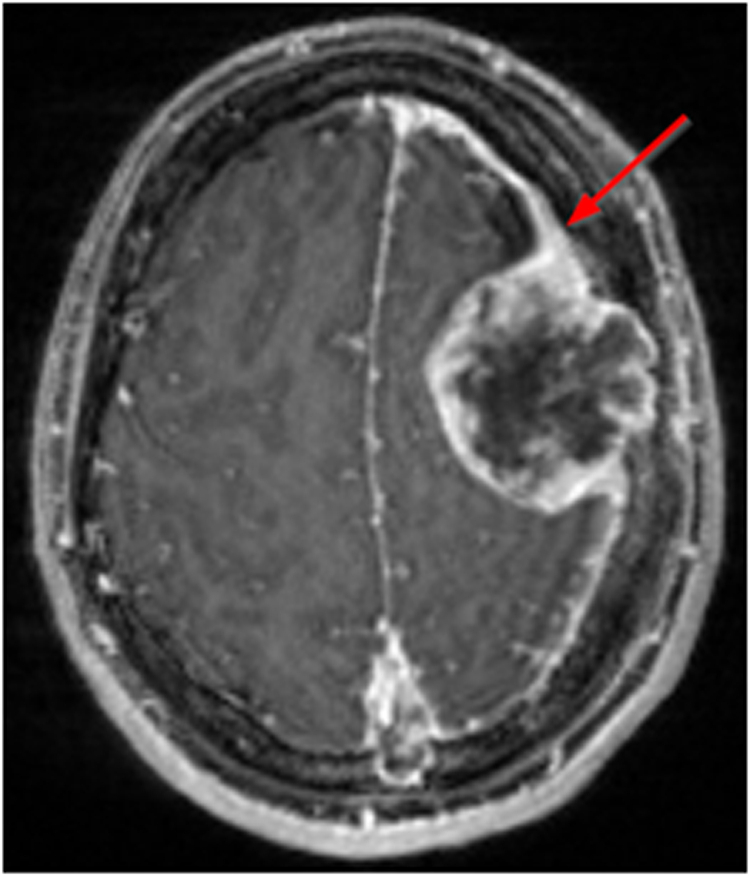
Example of an intracranial meningioma with a dural tail (red arrow) as shown on T1-weighted post-contrast MRI.

A key aspect of the BraTS Meningioma Pre-operative Dataset is the subdivision of the different tumor compartments that are visible on MRI sequences. The specific target volume delineation for intracranial meningioma MRI appearances and sub-compartments have been previously described^19–21^. In 2022, the Association des Neuro-oncologues d’Expression Francaise (ANOCEF) outlined consensus guidelines for meningioma gross tumor volume after 20 experts from 17 radiotherapy centers participated in a three round modified Delphi consensus^21,22^. The ANOCEF committee defined the enhancing gross tumor to include MRI T1 contrast-enhancing lesions, thickened meninges, and directly invaded bone^21^. This includes en-plaque meningioma and “dural tail” involvement, defined as thickening and enhancement of the dura infiltrating away from the lesion (Fig. 1)^23,24^. Non-enhancing tumor components include areas of mineralization or ossification typically with low signal intensity on T2-weighted imaging^24^ and cystic components with uniform low-signal intensity on T1-weighted imaging and high signal intensity on T2-weighted imaging^24^. Peritumoral edema, which appears as non-enhancing parenchymal T2/FLAIR signal hyperintensity surrounding the tumor, is present in 60% of meningioma cases and may be localized or extensive^25^.

**Fig. 2:**
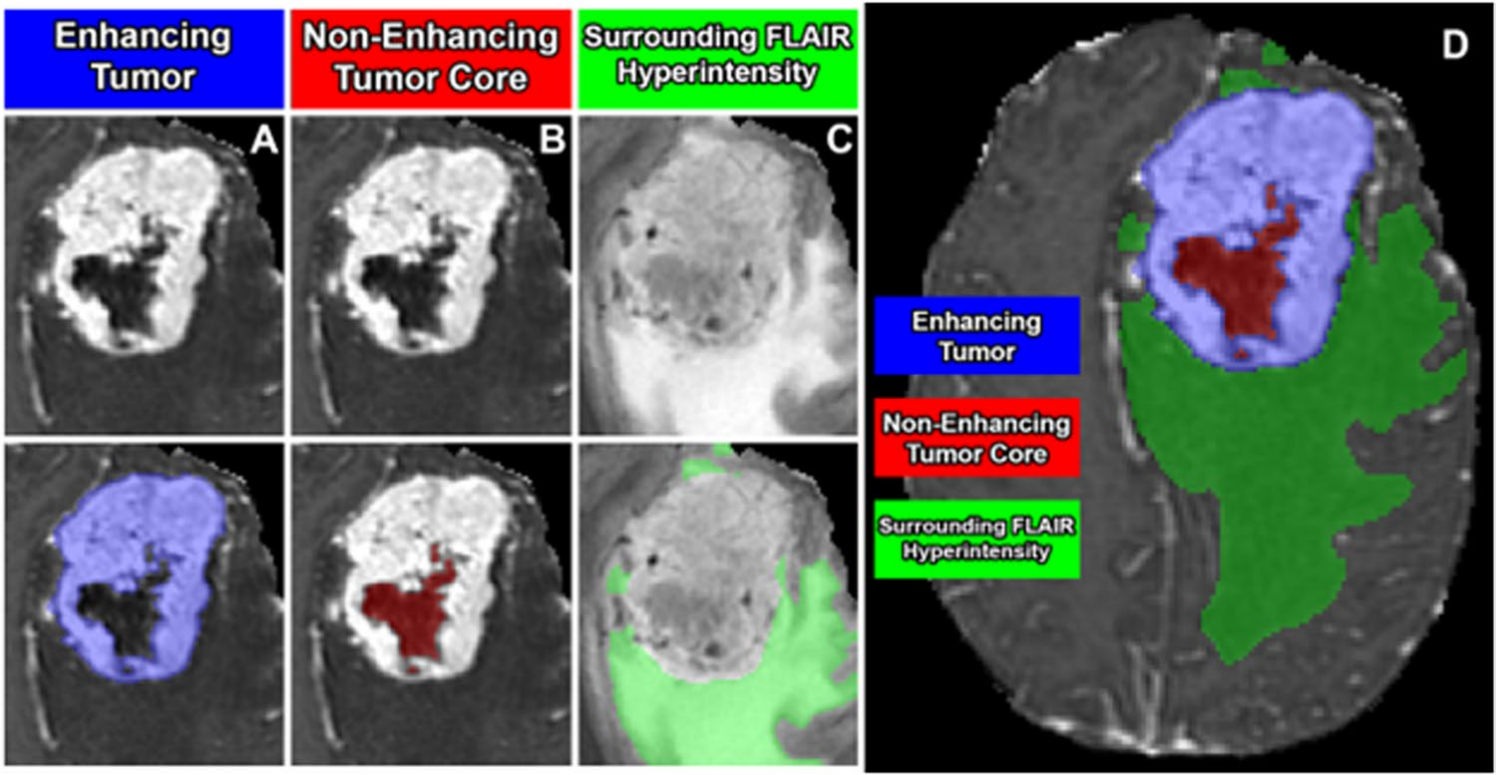
Meningioma sub-compartments considered in the BraTS Pre-operative Meningioma Dataset. Image panels A-C denote the different tumor sub-compartments included in manual annotations; (**A**) enhancing tumor (blue) visible on a T1-weighted post-contrast image; (**B**) the non-enhancing tumor core (red) visible on a T1-weighted post-contrast image; (**C**) the surrounding FLAIR hyperintensity (green) visible on a T2/FLAIR-weighted image; (**D**) combined segmentations generating the final tumor sub-compartment labels provided in the BraTS Pre-operative Meningioma Dataset.

Based on this prior work and others, we defined three distinct and non-overlapping tumor sub-compartments (Fig. 2)^19–21^. These include “enhancing tumor”, “non-enhancing tumor core”, and surrounding non-enhancing T2/FLAIR hyperintensity (SNFH). The enhancing tumor label included all contrast enhancing meningioma, focally thickened meninges (including dural tail), as well as en-plaque meningiomas. This label approximated the compartment of active, viable tumor, which would typically be targeted by radiotherapy. The non-enhancing tumor core label included all calcification, hyperostosis, necrosis, degeneration, cystic areas, and any other atypical non-enhancing tumor findings. This label along with the enhancing tumor label (together comprising the “tumor core”) corresponded to the portion of tumor related imaging abnormality that would typically be removed in a gross total resection. The SNFH label included the entire extent of tumor-related T2/FLAIR hyperintensity surrounding the tumor core. This label was distinct from the other labels in that it was composed entirely of brain parenchyma and was not expected to contain any tumor cells, but rather represented irritated, inflamed, and/or edematous brain tissue resulting from the adjacent tumor. Importantly, non-tumor-related brain parenchymal T2/FLAIR signal abnormality, commonly related to chronic microvascular ischemic white matter changes (e.g. leukoaraiosis) or other vascular pathology, was not included in the SNFH label.

### Automated meningioma pre-segmentation

Prior to manual correction, a deep convolutional neural network-based automated segmentation model was used for automated multi-compartment pre-segmentation. This model, implemented in nnU-Net (version 1) (https://github.com/MIC-DKFZ/nnUNet/tree/nnunetv1) was initially trained on a sample of 73 manually labeled studies from a single participating institution (UCSF). Of note, this initial sample consisted entirely of meningiomas that subsequently underwent surgical resection, which may bias the model to poorer performance for non-surgical meningiomas. During the manual correction phase of the challenge preparation, the automated segmentation algorithm was periodically retrained using additional manually corrected cases from other participating sites, including sites that contributed non-surgical meningioma cases. The purpose of iteratively retraining the model with new data was to improve its generalizability to different MRI appearances of meningioma and reduce pre-segmentation bias. Model weights for each of the different meningioma pre-segmentation models are publicly available at (https://github.com/ecalabr/nnUNet_models).

### Common errors of automated meningioma pre-segmentation

**Fig. 3:**
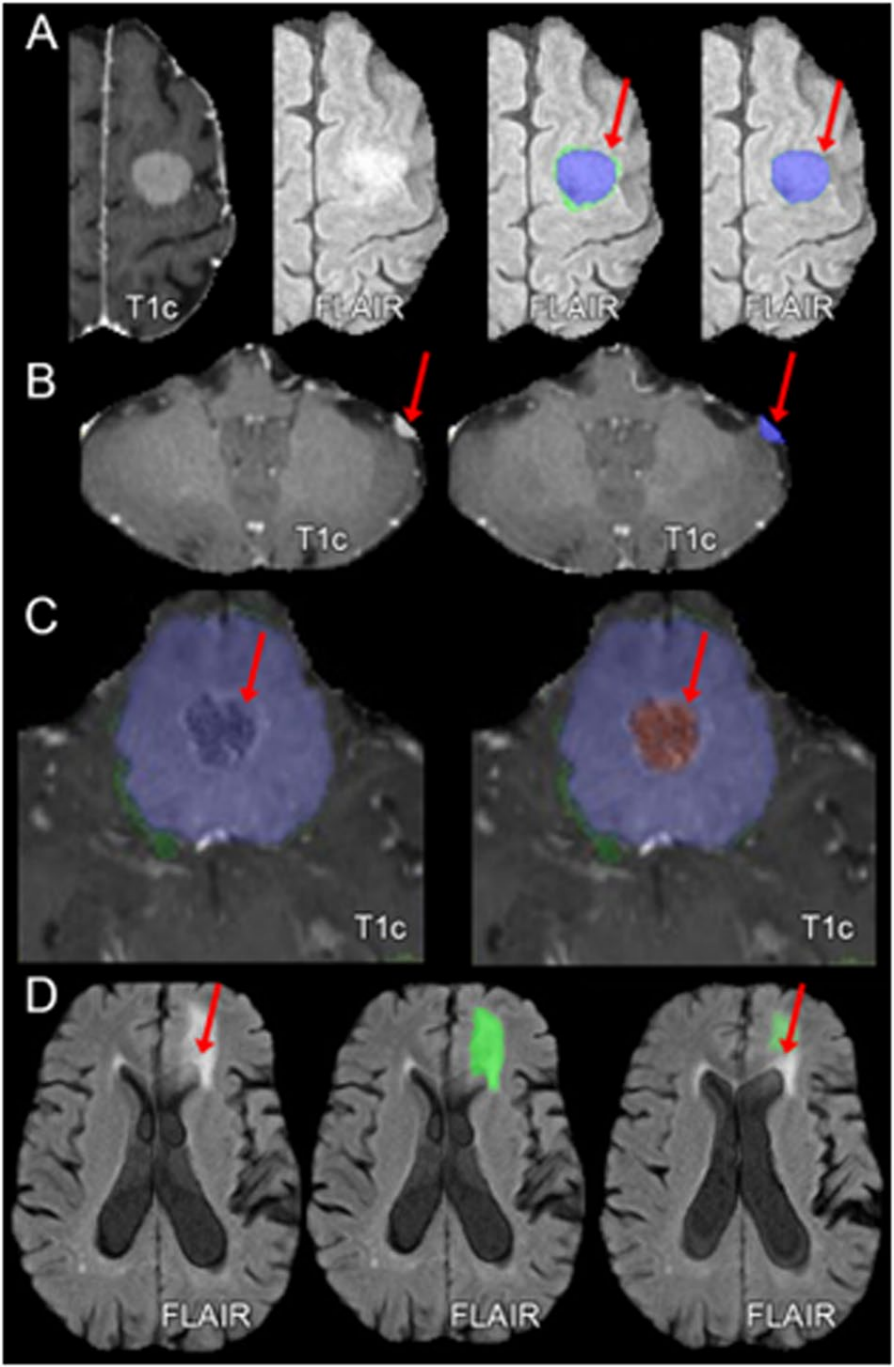
Examples of common errors of automated meningioma segmentation. (**A**) Erroneously marked a thin rim of edema that does not exist; (**B**) Missed small convexity meningioma; (**C**) Improper classification of non-enhancing tumor; (**D**) Tumor-related edema adjacent to presumed microvascular ischemic periventricular white matter FLAIR abnormalities. T1c: T1-weighted post-contrast imaging; FLAIR: T2-weighted fluid attenuated inversion recovery imaging.

Based on a subjective review of pre-segmented meningioma cases by dataset organizers, a set of commonly encountered automated segmentation errors were identified. The following list of commonly encountered errors was provided to dataset annotators in an effort to reduced inter-rater variability:

1. A thin rim of erroneously assigned SNFH label immediately surrounding smaller meningiomas without any true associated SNFH (Fig. 3a).
2. Incomplete or absent segmentation of small convexity meningiomas composed entirely of enhancing tumor, particularly when more than 1 meningioma was included in the field of view (Fig. 3b)
3. Improper assignment or incomplete segmentation of non-enhancing tumor compartments, including exophytic hyperostosis, cystic spaces, and areas of intrinsic T1 hyperintensity, which were sometimes erroneously labeled as enhancing tumor or SNFH rather than non-enhancing tumor core (Fig. 3c)
4. Inclusion of non-tumor-related brain parenchymal T2/FLAIR signal abnormality, most commonly chronic microvascular ischemic white matter changes (e.g. leukoaraiosis) within the SNFH label (Fig. 3d).

Examples of each of these errors of automated meningioma segmentation are provided in Fig. 3.

### Manual meningioma segmentation refinement process

For each meningioma case, manual review and refinement of pre-segmented labels was performed by individual volunteer “annotators” recruited from the ASNR society with widely varying experience levels spanning from medical students to fellowship-trained neuroradiologists. Subsequently, manually corrected annotations were reviewed by a single board-certified neuroradiologist “approver” (author EC). Manual corrections were performed using ITK-SNAP, a free, open-source, multi-platform software application used to segment structures in 3D biomedical images^26^. Annotators were provided with each of the following: 1) basic instruction on using ITK-SNAP for meningioma segmentation, 2) written descriptions of the composition of each tumor sub-compartment, and 3) a list (with examples) of common pre-segmentation errors to identify and address (similar to Fig. 3). In cases where manually corrected segmentations were deemed inaccurate, they were returned to the annotator pool for further corrections and re-review. This process was repeated until the segmentations were deemed accurate.

## Data Records

The BraTS Meningioma Pre-operative Dataset training (1,000/1,424, 70%) and validation (141/1,424, 10%) data are publicly available on Synapse^14^. The testing dataset (283/1,424, 20%) will be kept private for the foreseeable future to allow for the unbiased assessment of future segmentation algorithms. The “Meningioma supplementary clinical data and imaging parameters for training and validation sets.xlsx” file on the Synapse data repository describes the case level clinical patient data and the image parameters for the training and validation cases^14^. The supplementary file “Meningioma Dataset Access Steps” provides step by step instructions on how to access the data.

## Technical Validation

### Patient clinical and demographic data

All clinical characteristics of the subjects included in the BraTS meningioma collection were obtained from clinical records from each respective academic institution without specific disclosure of the data collection method from each institution. This approach was taken to encourage data contribution. Clinical data included patient sex and age at time of the diagnosis, as well as CNS WHO grade. Specific information regarding patient pre-operative treatment was not included as part of data collection. No additional validation of the raw clinical data was conducted in the BraTS meningioma collection.

### Image pre-processing and skull stripping

All pre-processing steps were manually reviewed by a fellowship-trained neuroradiologist (author EC) to ensure proper co-registration to the SRI24 atlas space, image quality, adequate skull stripping, presence of an intracranial meningioma, and absence of a non-meningioma intracranial tumor. Any pre-processing errors were manually corrected before inclusion in the dataset. For a majority of exams, original, unprocessed x and y image resolution and slice thickness were available and are included on the Synapse data repository^14^. Additional original image metadata was either not available, not approved for public release by governing data use agreements, or intentionally witheld to prevent BraTS challenge participants from fingerprinting exams from specific sites. However, data regarding site of origin for each exam is available and can be shared by request to the corresponding author on a case-by-case basis.

### Meningioma segmentations

All manually corrected meningioma segmentations were manually reviewed by a board-certified neuroradiologist “approver” (author EC) following the established annotator/approval model used in prior BraTS challenges^7^. In cases where the approver identified an inaccurate or incomplete segmentation, the case was returned to a different annotator for further refinement with notes indicating remaining issues. This process was repeated, if necessary, until the segmentations were deemed accurate.

### Supplementary information

Meningioma Dataset Access Steps

## Data Availability

In line with the scientific data principles of findability, accessibility, Interoperability, and reusability^27^, the tools used throughout the generation of these data are publicly available. Specifically, we used the FeTS toolkit [FeTS] to perform all pre-processing steps, including co-registration, and skull stripping, which is publicly available at (https://fets-ai.github.io/Front-End/)^17^. The nnU-Net model as used for initial pre-automated segmentation is publicly available at (https://github.com/ecalabr/nnUNet_models).
