# Supplementary material for: A multi-institutional meningioma MRI dataset for automated multi-sequence image segmentation
Source: Sci Data. 2024 May 15;11:496. doi: 10.1038/s41597-024-03350-9 (PMC11096318; doi:10.1038/s41597-024-03350-9)

This document outlines the steps needed to freely access the BraTS Pre-operative Meningioma Dataset.

1. Click on either of the following links, which will direct you to the Synapse hosting site.
   1. <https://doi.org/10.7303/syn51514106>
   2. <https://www.synapse.org/#!Synapse:syn51514106>
2. Click on the “key” symbol next to “Access” to login to Synapse.


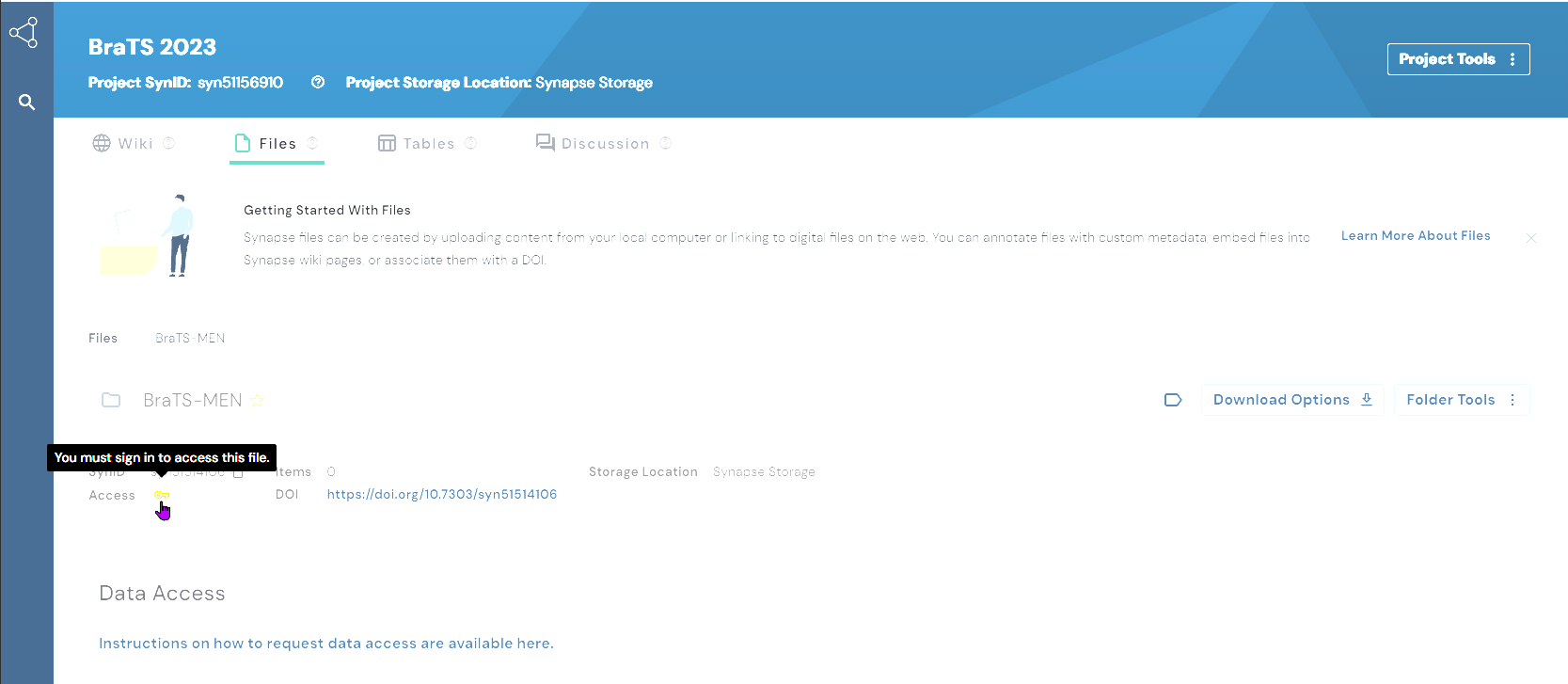


1. Either login to your previously created account or create an account.


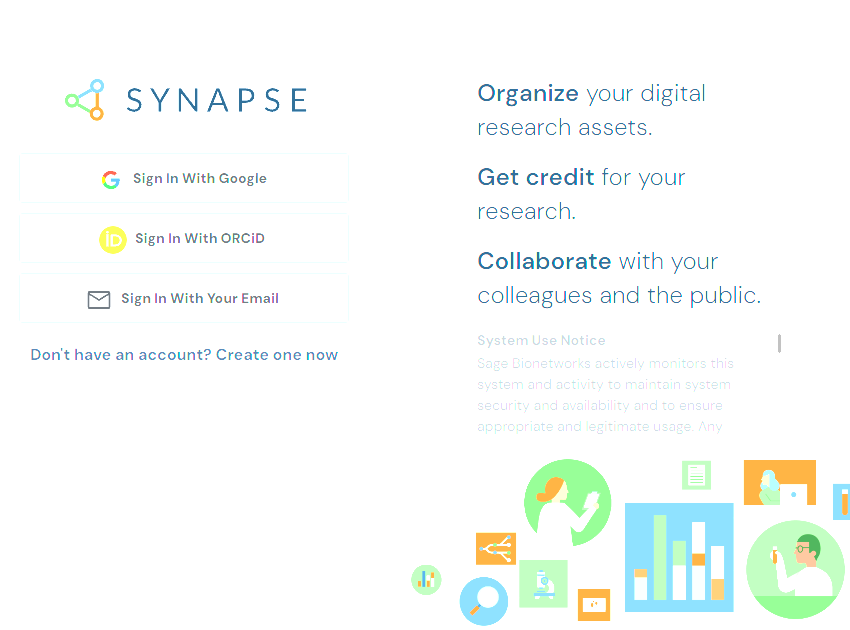


1. After logging in, the “key” will change to a green “unlocked” symbol.


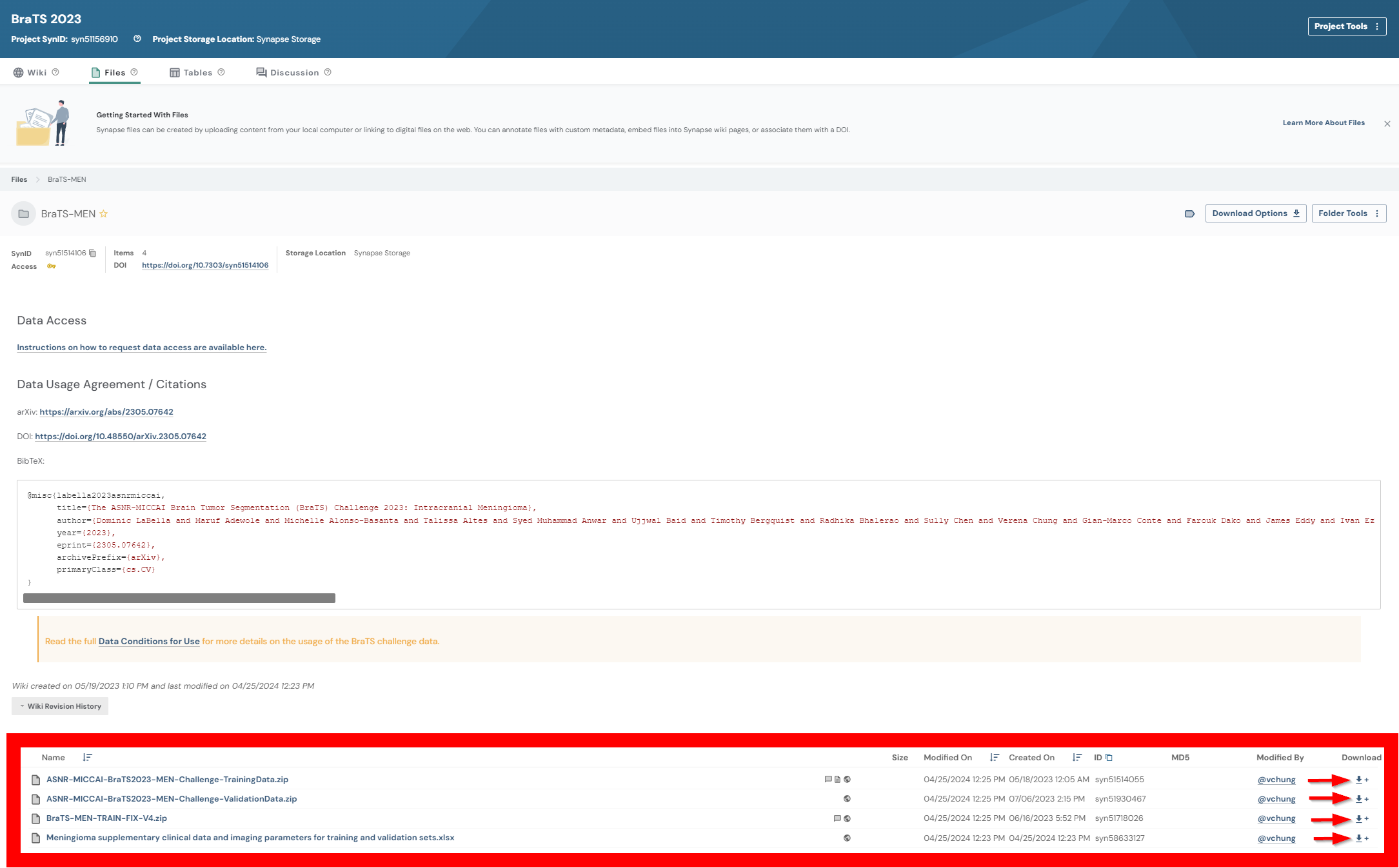


1. Click on the download button for any of the respective folders. Then, click on the “View Download Link”.


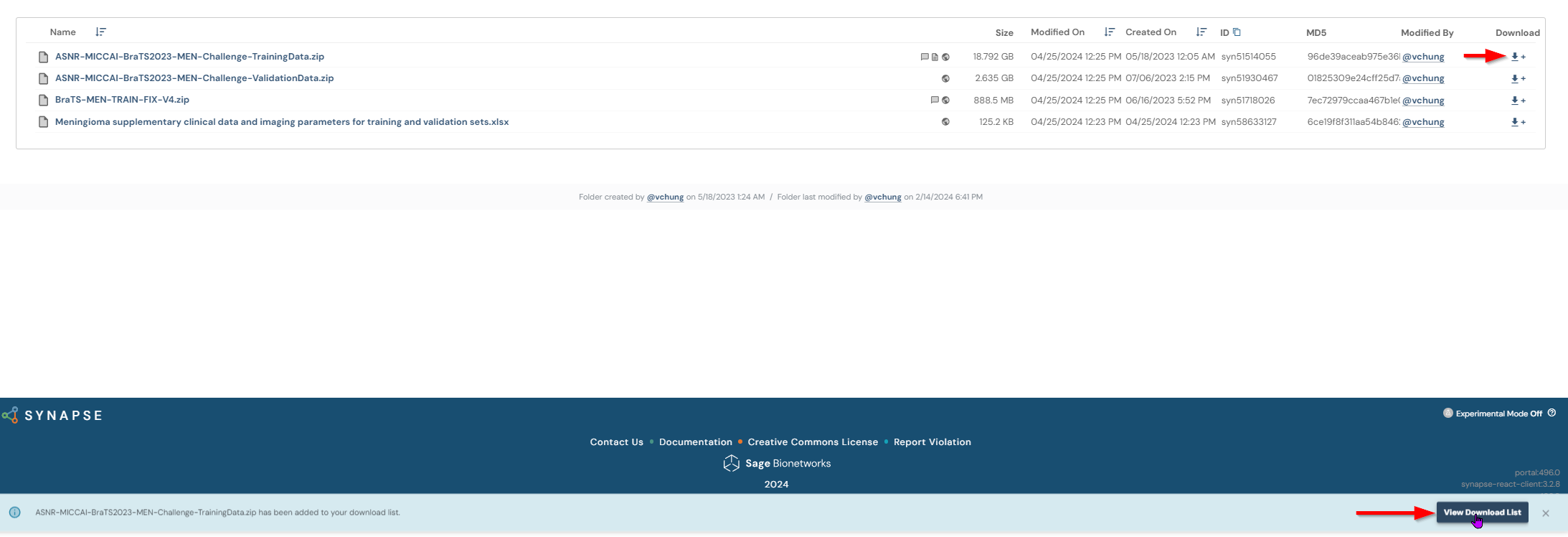


1. This will take you to the download cart. At which point you can click on the download button to download the data.


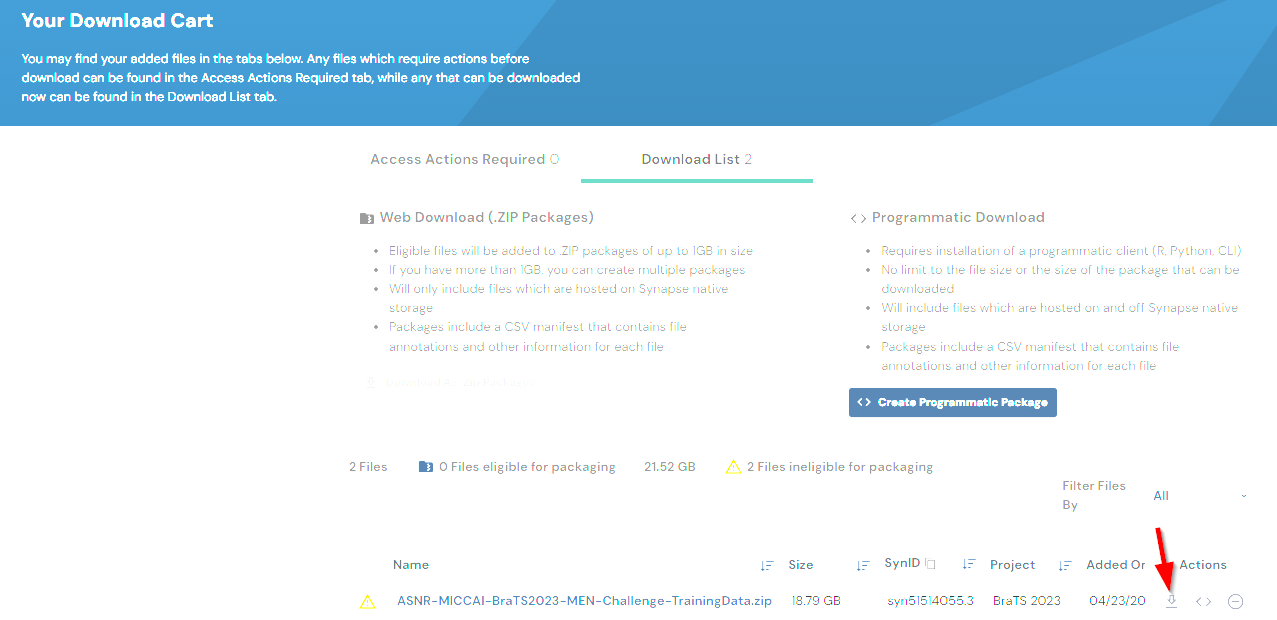

Supplement: Supplementary file 1 — Meningioma Dataset Access Steps [file 41597_2024_3350_MOESM1_ESM.docx]
